# Evidence of embodied social competence during conversation in high functioning children with autism spectrum disorder

**DOI:** 10.1371/journal.pone.0193906

**Published:** 2018-03-05

**Authors:** Veronica Romero, Paula Fitzpatrick, Stephanie Roulier, Amie Duncan, Michael J. Richardson, R. C. Schmidt

**Affiliations:** 1 Center for Cognition, Action and Perception, Department of Psychology, University of Cincinnati, Cincinnati, OH, United States of America; 2 Department of Psychology, College of the Holy Cross, Worcester, MA, United States of America; 3 Department of Psychology, Assumption College, Worcester, MA, United States of America; 4 Department of Developmental and Behavioral Pediatrics, Cincinnati Children’s Hospital Medical Center, Cincinnati, OH, United States of America; 5 Department of Psychology and Perception in Action Research Centre, Faculty of Human Sciences, Macquarie University, Sydney, NSW, Australia; Harvard Medical School, UNITED STATES

## Abstract

Even high functioning children with Autism Spectrum Disorder (ASD) exhibit impairments that affect their ability to carry out and maintain effective social interactions in multiple contexts. One aspect of subtle nonverbal communication that might play a role in this impairment is the whole-body motor coordination that naturally arises between people during conversation. The current study aimed to measure the time-dependent, coordinated whole-body movements between children with ASD and a clinician during a conversational exchange using tools of nonlinear dynamics. Given the influence that subtle interpersonal coordination has on social interaction feelings, we expected there to be important associations between the dynamic motor movement measures introduced in the current study and the measures used traditionally to categorize ASD impairment (ADOS-2, joint attention and theory of mind). The study found that children with ASD coordinated their bodily movements with a clinician, that these movements were complex and that the complexity of the children’s movements matched that of the clinician’s movements. Importantly, the degree of this bodily coordination was related to higher social cognitive ability. This suggests children with ASD are embodying *some* degree of social competence during conversations. This study demonstrates the importance of further investigating the subtle but important bodily movement coordination that occurs during social interaction in children with ASD.

## Introduction

Children with Autism Spectrum Disorder (ASD) exhibit persistent impairments in social communication and social interactions that last throughout their lives [1]. These impairments are found even in relatively high functioning children and include an inability to display or reciprocate appropriate verbal and nonverbal aspects of communication and, in turn, successfully carry out and maintain effective social interactions in multiple contexts [1–6]. These deficits can severely impede learning and overall social functioning at home and in school, as well as make treatment difficult. Historically, the research and assessment tools used to identify and understand the social interaction deficits present in the ASD population have centered on qualitative measures of the semantic, pragmatic and nonverbal qualities of communication. As important as it is to understand what is being said during face-to-face interaction, words are not dissociated from a body which moves according to what is being said sometimes subtly [7,8]. One such aspect of subtle nonverbal communication is the whole-body motor coordination that naturally arises between people during conversation.

An ever growing body of literature (for a review see [9]), has shown that language and whole-body motor coordination are intertwined and both are very important to achieve successful social interactions. This research has found that the subtle and sometimes non-obvious aspects of social interaction are fundamental for maintaining a communicative exchange and promoting interpersonal responsiveness and verbal comprehension [10–13]. In fact, research has shown that simply coordinating one’s movements with another actor can influence rapport, feelings of connection and feelings of social competence [10–13]. Past research has also found that deficits in social motor coordination are associated with psychological dysfunction such as schizophrenia [14–16] and borderline personality disorder [17]. As shown by this research, when communicating, people do so through their body movement as much as through what they say, in other words, communication is embodied, and therefore the body movements of actors should not be ignored. Even though social motor coordination is considered to be an integral part of maintaining successful social interactions [12,18], it has until recently been overlooked in ASD research. It is therefore important to understand the degree to which social motor coordination (or the lack thereof) is related to social competency in children diagnosed with ASD.

The research investigating social interaction impairments in ASD has so far been limited and often contradictory and mostly focused on understanding break-downs in particular aspects of motor production and understanding. For example, a number of studies have found that children with ASD have difficulty imitating and using gestures during conversations [19–22], difficulties planning an action [23,24] and problems executing a motor plan [24–26]. Additionally, research has found that children with ASD struggle with understanding someone else’s intention when watching them performing a motor act [26,27]. One limitation of this research is that it has been done through the use of static pictures and asking children to explain their understanding verbally. More importantly, there has been little distinction between measuring whether children with ASD *can successfully perform* different parts of social interactions versus *how* they carry out these interactions [28]. We would like to focus our attention on the latter.

In general, researchers have recently found that people with ASD perform body movements differently than their typically developing counterparts [23] and that they are generally more variable even when they are ultimately successful in the task [23,28–30]. Moreover, when looking at the coordination between children and their parents, typically developing children have been shown to spontaneously modify their movements more than children with ASD to achieve this desired coordination [12]. Additionally, some research reveals major differences in the way children with ASD and typically developing children imitate and synchronize with an experimenter in various social tasks [28,30–32]. Specifically, even when asked to reproduce simple sequences of motor movements in time with an experimenter, children with ASD showed significantly lower levels of motor coordination than children without ASD. However when children were asked to just repeat the sequence after the experimenter performed it (without having to synchronize their movements), the differences in motor coordination were attenuated [28,30–32].

Based on the important role that social motor coordination plays in achieving successful social interactions, the current study aimed to measure the degree of whole-body motor coordination that arises between a child previously diagnosed with ASD and a clinician during a conversational exchange. The approach used in this study draws from the tools of nonlinear dynamics that enable an analysis of time-series data. In our case, we used the time-dependent, coordinated whole-body movements of participants [33,34]. Time series data involves recording a sequence of successive measurements taken at an equally space time-interval. This technique generates a large number of data points from a single source, without the need of a human observer. The behavioral coding typically used in psychological research involves having a researcher observe a particular behavior and summarize the behavior into specific categories or codes based on a pre-determined set of rules and usually requires several observations from many different sources. Time series data is therefore advantageous because it generates a very large number of data points from one source and offers an objective measure of behavior without the need for subjective coding by a researcher.

The techniques selected here were chosen to provide a dynamical measure of the patterning of whole-body movement variability for each participant and the clinician during a conversation (fractal analysis: [35–38]), as well as the degree of whole-body coordination between them (cross-wavelet: [8,39]). Such tools will provide a quantitative and objective measure that has the potential to further our current understanding of ASD in general and strengthen diagnostic power in the future [28,31,32]. The social problems evident in ASD are pervasive and involve many aspects of social interaction and communication, including but not limited to, gesturing, planning and executing movements, maintaining eye contact, engaging in restrictive and repetitive behaviors. While there is value in understanding which specific dimensions of social interaction are impaired, social interactions are dynamic, holistic events that involve complex interactions among all the various components. As such, the success of social interactions cannot be reduced to one specific dimension of the interaction. Therefore, for the current study we were interested in measuring the implicit and unconscious whole-body movement coordination and variability that arose during conversational exchanges, rather than focusing on only one mode of social motor communication.

Furthermore, the current study aimed to situate these newer analysis methods in context with traditional measures of social cognition used in the ASD literature. Previous research shows that children diagnosed with ASD lack what is commonly referred to as theory of mind (ToM), which means that they have difficulty understanding other people’s thoughts, beliefs, and perspectives [40]. ToM is tested by asking children to answer a series of hypothetical questions regarding what someone else (sometimes represented by a puppet or doll) would feel like or do in the described situation [40] (for a review of past findings see [41]). Some researchers have claimed that lack of ToM does not explain all deficits present in ASD, is not unique to ASD, and is not universally experienced by all individuals with ASD [42,43]. However, ToM remains a central measure used in the ASD literature to classify children [41,44,45] and there is some consensus that there is evidence that the development of ToM is delayed in children with ASD and the attribution of mental states is challenging for individuals with ASD, even if they do pass ToM tests [43]. The current study employed four ToM tasks, three that required verbal answers and one non-verbal measure. The scores for all four ToM tests were then combined and the relationship between it and the dynamic social motor coordination measures were tested.

Another skill that children with ASD struggle with is social attention. Dawson and colleagues [46] argue that children with ASD attend to the social environment in a different way than children without ASD. One critical component of social attention in ASD is joint attention (JA). Joint attention refers to the ability to orient attention based on the eye gaze of another person with whom one is interacting [47–49], and it is a skill that typically developing children (TD) seem to be able to master at 12 months, while most children with ASD show impairments [46]. Furthermore, JA is a skill that has been shown to predict language development and autism severity [46,50] and when targeted in early intervention programs it has shown positive outcomes [48]. Due to the central role that JA plays in diagnosing and treating ASD, it was important to investigate the relationship that might exist between this skill and the dynamic whole-body motor coordination measures introduced in the present study.

Finally, to place the current study into the broader ASD research context, the relationship between the dynamic motor coordination measures and the scores obtained from one of the “golden standard” diagnostic tests was investigated. The interpersonal coordination measures described in the current study were obtained from one task that is part of the Autism Diagnostic Observation Schedule, Second Edition (ADOS-2, [51]) battery. It was therefore important to investigate the relationship between these new measures and the scores that the children were assigned on the different subscales of the ADOS-2 (Social Affect and Restricted and Repetitive Behavior) as well as their overall score on the test.

As Tager-Flusberg argued [43], no single impairment has been sufficient to understand the heterogeneity of the core features of ASD. This is not surprising since social interactions and communication involve a complexity of implicit and explicit factors—knowing what to pay attention to and when, understanding the perspective of other people, and controlling the body to produce speech, gestures, and joint actions. Therefore, a fuller understanding of the inter-relationships between various implicit and explicit aspects of social functioning is worthwhile and could provide us with new insight into the range of social and communication behaviors evident in ASD. Here, we tested the associations between the dynamic whole-body motor movement measures introduced in the current study and the measures used traditionally to categorize ASD impairment (ADOS-2, joint attention and theory of mind). A lack of significant relationships between these traditional measures and whole-body social motor movement would suggest they are independent processes. If these relationships are indeed found, it suggests that these motor measures may be tapping into fundamental aspects of social interaction that are also captured by traditional social cognitive measures that assess children’s understanding of social situations. Significant relationships between the measures would either suggest they may share underlying processes or a disruption in one (or more) may lead to a disruption in another.

In addition, our approach, taps into the on-line social-motor processing that takes place during a social exchange, and assesses the degree of whole body coordination, rather than discrete behavior of task success or failure. Such an approach holds much promise for understanding the continuum of social and communication behaviors observed in ASD and provides an evaluation of the *degree* of embodied social cognition evident in ASD. Of particular interest is whether the level of social embodied cognition is related to the level of social cognition impairment.

## Method

### Participants

Data used for this investigation was part of a larger study [32] in which there were 50 high-functioning children with ASD who were diagnosed by a licensed clinical psychologist or medical doctor based on DSM-IV_TR criteria [52] and confirmed by the Autism Diagnostic Observation Schedule, Second Edition (ADOS-2, [51]) module three. Five of the children in the ASD group were classified as non-spectrum and were eliminated from the analysis. Due to analysis limitations explained below, video clips of only 28 of the remaining 45 participants were used in this study. There were 25 males and 3 females. Of this sample of children, 23 were white, 2 were African American, 1 was Asian, and 2 were multi-racial. Other general information about the group of participants can be found in Table 1, such as chronological age in months of the participants, ADOS-2 scores and sub scores, Joint attention scores and Theory of mind scores. Additionally, to help put the sample in context, verbal and non-verbal age in months was calculated based on scores obtained from the Differential Ability Scales, Second Edition (DAS-II) to categorize cognitive ability. Finally, scores obtained from the Clinical Evaluation of Language Fundamentals (CELF) are reported. Specifically, the sub tests of Following Directions (FD) and Formulated Sentences (FS) in order to categorize the sample’s language abilities at the time of testing.

**Table 1:** General information about the sample.

	Mean	Standard Deviation	Range
**Chronological age**	102.71 months	16.29 months	72–129 months
**DAS verbal mental age**	98.09 months	23.63 months	62.5–153 months
**DAS non-verbal mental age**	106.57 months	25.32 months	61.75–152.5 months
**ADOS social affect (SA) score**	8.93	4.08	1–17
**ADOS RRB score**	2.57	1.83	0–7
**ADOS comparison score**	6.57	2.32	1–10
**CELF FD score**	7.23	3.92	1–14
**CELF FS score**	7.96	3.95	1–16
**Joint Attention score**	0.737	0.275	0.25–1
**Theory of Mind score**	3.136	0.938	0.667–4

All parents of participants gave informed, written consent for their children to take part in the study, and releases were also obtained for the video recordings. Participants received a $100 gift certificate for participating in the study. The project was approved by the Cincinnati Children’s Hospital Medical Center’s (CCHMC) Institutional Review Board and participants were recruited from CCHMC and local communities through print advertising, a recruitment brochure, email, social media, and community events.

### Materials and procedure

Video recordings of the conversation and reporting task of the administration of the ADOS-2 module 3 (task 8) was used to measure the whole-body movements of the clinician and the children during conversation. The task is normally initiated by the clinician and allows the examiner to observe the child’s ability to participate in a natural conversation, usually about an event or memory. The clinician uses this task to assess the child’s general conversational skills, such as ability to elaborate or build on statements, appropriate use of gestures and facial expressions, and ability to lead or continue the conversation. Our analysis of this task, however, focused on analyzing the body movements of the child and clinician during these conversations. The clinician did not know at the time of the ADOS administration that their body movements and the movements of the child would be measured. Such body movements during conversation tend to be largely unconscious. This task usually follows a comment given by the child during any of the tasks in the ADOS-2 and is followed by a prompt from the clinician for more information regarding the topic brought up by the child and can therefore occur during any point during the ADOS administration. These conversations are mostly child-directed, in the sense that the child focuses on things that he/she enjoys talking about, such as a favorite video game or vacation. Examples of the kinds of conversations that develop between the child and the clinician can be found in Appendix A. As can be seen from these examples, the clinician mostly responds to the information being provided by the child about a topic he/she is interested in. When appropriate the clinician tries to elaborate on the topic the child has selected for conversation to continue the interaction.

Video segments of only the conversation and reporting task (task 8) were selected because both the child and clinician were involved in the conversation, were sitting across from each other at a desk, and did not use manipulatives (which could interfere with naturally-occurring conversational body movements). Depending on the child’s performance during the first administration of the conversation and reporting task, the clinician will typically administer the task multiple times throughout the administration of the ADOS assessment. For our analysis, we focused only on the first administration of the conversation and reporting task to standardize across participants due to the fact that some children only performed the conversation and reporting task once. We chose this one task from the ADOS administration because neither of the participants crossed the midline of the screen, which was important given the automated nature of the video analysis performed (see Video Analysis below). It is important to note that *no other* structured conversations that are part of the ADOS-2 module 3 assessment (e.g. description of a picture, telling a story from a book) were analyzed.

The original dataset also consisted of a number of social cognitive and language ability measures and the two measures explained below were used here to evaluate the relationship between social cognition and the social whole-body movements of the clinician and child. Responding to Joint Attention (RJA) was assessed by using the gaze-monitoring task [44,53]. In this task, the experimenter focused her gaze on an object in the room and asked the child “what am I looking at?” The child received 1 point for each correct fixation on the picture or object the examiner looked at, for a total of 4 possible points and the children’s scores can be found in Table 1. Children also completed four theory of mind (ToM) tasks—three verbal and one non-verbal—to measure understanding of intentionality. The “Smarties” candy task [54,55] was the first verbal task and the child received 1 point for each false belief, reality, memory, and justification question answered correctly, for a total of 4 points. The Contents False Belief task [56] was the second verbal task. The child received 1 point for each question answered correctly for a total of 2 points. In the “Sally-Ann” theory of mind task [40] children received 1 point for each correct answer on the false belief, reality, and memory questions, for a total of 3 points. The choose-a-drawing task [57] was used as the non-verbal task. The child received 1 point for each false belief and reality question answered correctly, for a total of 2 points. The sum of all of ToM tasks (out of a possible 11 points) was calculated. Due to the lengthy experimental procedure in the original study, the experimenter did not always ask each child all 11 questions. Therefore, a mean was calculated for each of the four tasks (number correct/total number of questions for each task) and the sum of the mean for each ToM task was calculated and used as the ToM total score (out of a possible 4 points) in the data analysis, and the sample’s scores can be found in Table 1.

The social affect subscale, the restricted and repetitive behavior subscale, and the comparison score from the ADOS-2 were also used in the analysis. The comparison score of the ADOS-2 is derived from the child’s total score and their age in years and is to put the severity of their symptoms in better context.

### Video and data analysis

**Fig 1:**
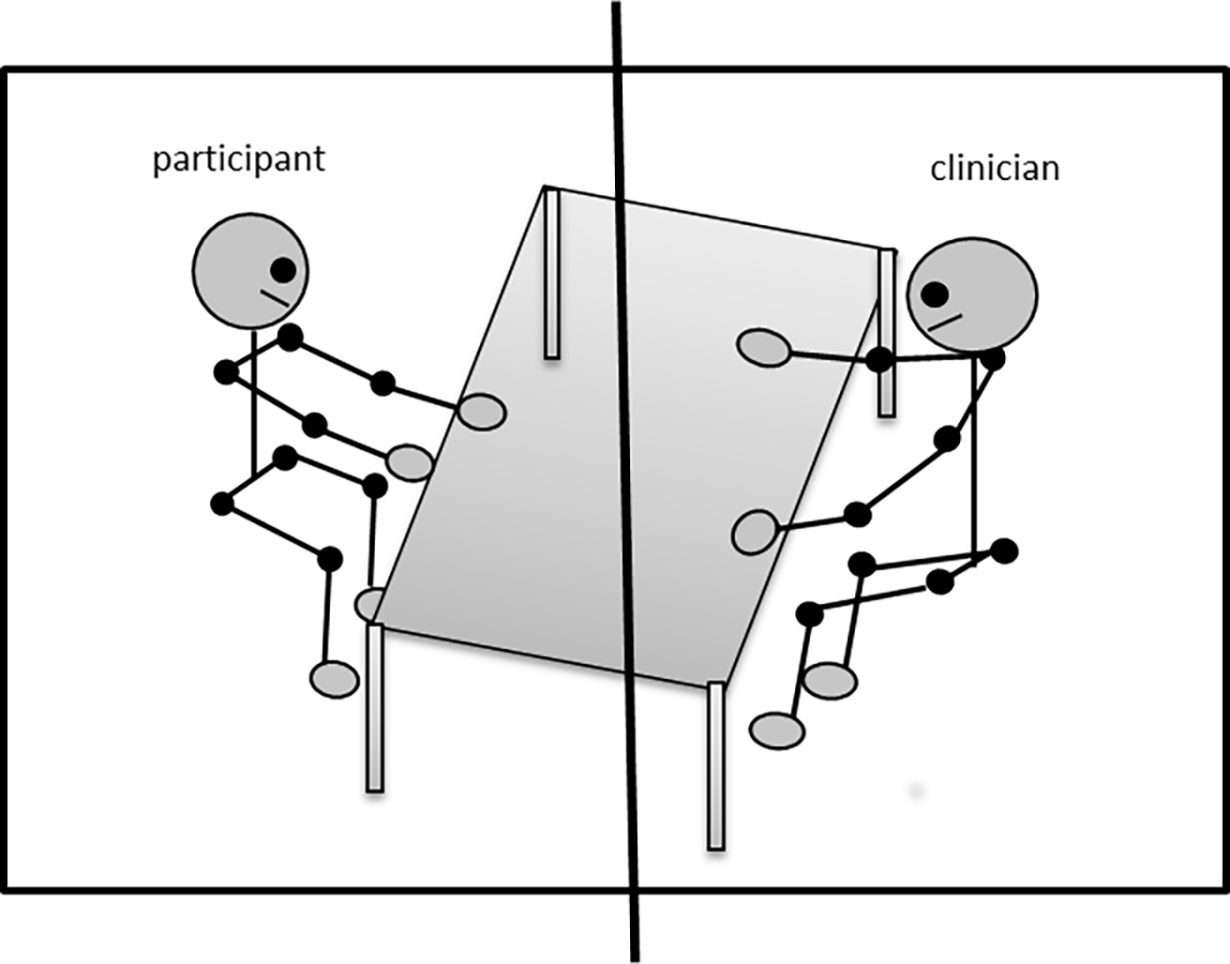
Example set-up and video frame. General set-up of the room in which the ADOS-2 administration took place. Children sat across from a clinician with a table between them. The segments included in the analysis were carefully chosen such that the child always stayed in his/her half of the video frame and did not cross the middle of the frame (depicted in this Fig as a thick black line). The clinician also stayed in her half of the frame for the duration of the conversation that was analyzed.

Whole-body movement in the conversational interactions was measured through a split-screen analysis of the recorded videos. Out of the 45 possible video clips in the original sample, only 28 met our criteria that neither participant crossed the midline, therefore the analysis of 28 videos is reported here (average length 187 s, range: 78–380 seconds). For this analysis, the amount of pixel change between adjacent video frames was measured from 30Hz video recordings. This provided us with a very dense and robust measurement of whole-body movement that consistent of an average of 6, 292 data points for each participant’s movement during the conversation and reporting task (range 2818–11, 393 data points). We were able to obtain separate whole-body movement measures of the child and examiner because they were situated on separate halves of the screen as can be seen in Fig 1. The amount of pixel change between adjacent video frames of each side of the frame corresponds to the amount of whole-body movement of a participant at that moment if they are the only source of movement in that part of the frame, in other words, if no movement occurs in the background [16,58,59]. This method has been used to capture gross whole-body movement in different contexts [8,16,30,58,59], including children with ASD performing different rhythmic tasks [30]. In fact, it has been shown that this method is able to capture coordinative patterns that arise between co-actors to a similar extent as more expensive motion capture equipment such as the Polhemus [30]. A main advantage of this method of extracting whole-body motor movement is that it is completely wireless. Given the hypersensitivity exhibited by many children with ASD, not having to attach any motion tracking equipment to their limbs was helpful. Additionally, this method is completely automatic and did not involve the coding of specific types of movements, such as gestures. The benefit of this is that it gives a measure of the social interaction by including movement from all the different movement categories.

#### Cross-wavelet analysis for degree of coordination

**Fig 2:**
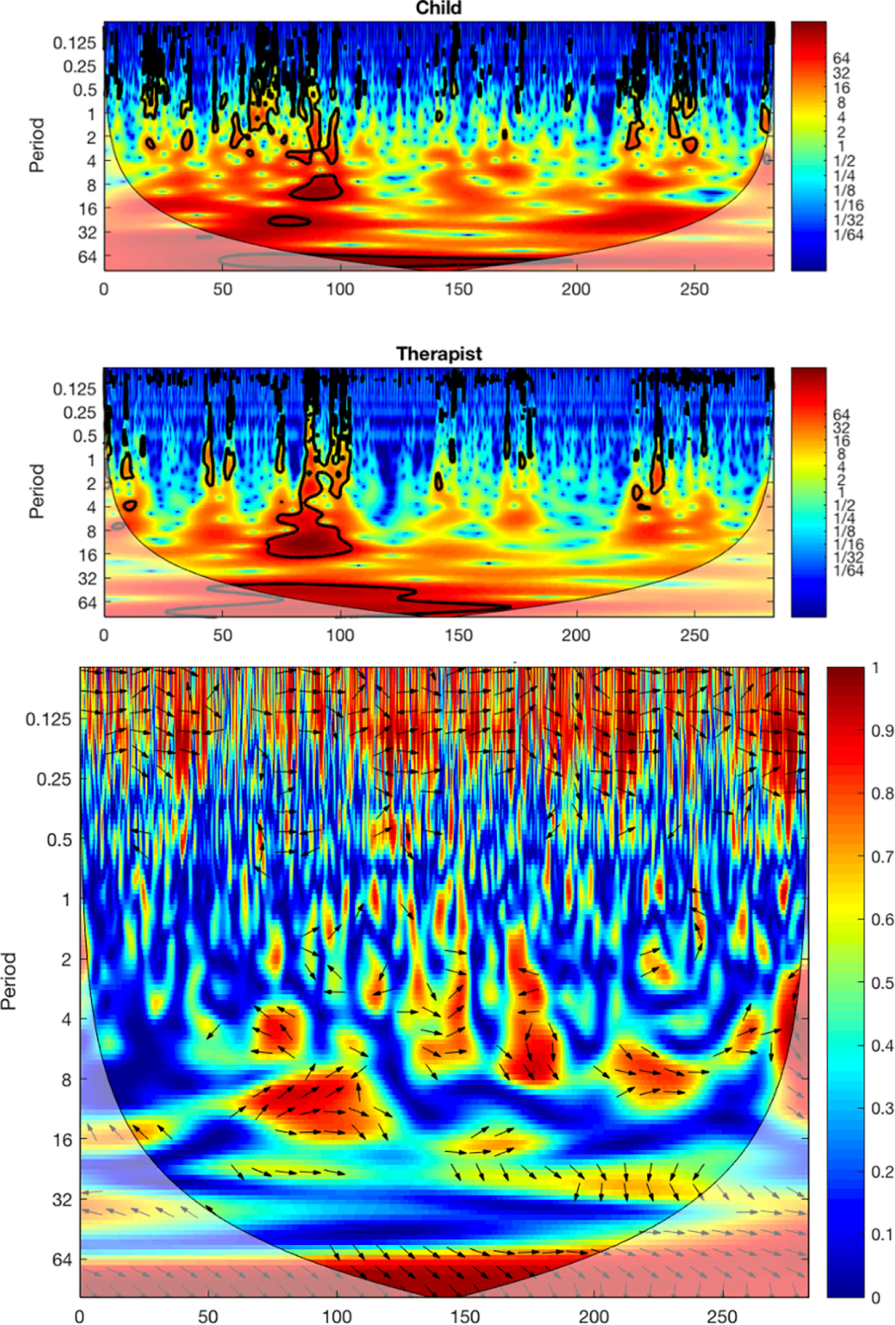
Example cross-wavelet plots. Wavelet power of child (top), clinician (middle) and cross-wavelet coherence (bottom) plots for an exemplary interaction. The length of the interaction was 283 s. In the top two plots, the color indicates the degree of spectral energy at different points in time of the interaction (x-axis) and at different time scales (y-axis periods). In the bottom plot, coherence magnitude and relative phase at a given time scale and a point in time are denoted by color and the orientation of the arrow (pointing right: child-clinician inphase whole-body movement coordination; left: child-clinician antiphase whole-body movement coordination; down: child leading by 90°), respectively.

The whole-body movement time series of the child and the clinician were analyzed for degree of social movement coordination between them. Because motor coordination in social interactions seems to have intermittent, nested periodicities [8,59], a cross-wavelet method of spectral decomposition was used to assess the degree and pattern of the participants’ movement coordination at individual time scales as they change across an interaction. A wavelet analysis is a time-frequency based analysis that allows one to perform a spectral decomposition continuously across time so that an estimate can be made for how the spectrum changes for each point in time [39,60]. It is useful for complex time series with non-stationarities such as these time series of the participants’ whole-body movements during conversation. Wavelet plots (see Fig 2, top) display the amount of power (indicated by colors where blue is low power and red is high power) at each time scale (y-axis) for each point in time (x-axis) of the trial. A cross-wavelet analysis evaluates the cross-spectrum of two time series across time, and hence, can uncover how the time-localized coherence and relative phase at different frequency ranges (time scales) changes across the course of a trial. A cross-wavelet coherence plot (see Fig 2, bottom) displays the coherence (correlation between the child and clinician indicated by colors where blue is low correlation and red is high correlation) at each time scale (y-axis) for each point in time (x-axis) of the trial.

In our analyses, we used a Morlet wavelet of order 8 to evaluate the coherence and relative phase at fifteen time scales from .5 s to 10 s, which are time scales associated with both gesturing and turn taking during conversation [61]. The circular mean of the relative phase and the mean of the coherence at these subsidiary time scales were extracted to evaluate pattern and degree of body motor coordination, respectively, and submitted to statistical analyses. In order to evaluate whether pattern and degree of body motor coordination at the different time scales were different from that expected by chance “virtual pairs” were created to form control condition estimates. These virtual pairs were obtained by combining the time series of the child in a session with the times series of the clinicians from another session. When the time series were of unequal lengths, the longer time series was truncated to equal the length of the shorter time series because the analysis required time series of equal length. The analysis of these virtual pairs provided estimates of chance motor coordination that may occur between individuals when they were not affecting one another’s movements.

#### Fractal and multi-fractal analysis to investigate variability in the movement

To evaluate the variability structure of the bodily movements of each of the participants, fractal and multifractal analyses were performed on both the child’s and clinician’s whole-body movement time series. Detrended fluctuation analysis (DFA) [62] and a multifractal detrended fluctuation analysis (MFDFA) [63] were used. DFA generates a scaling (Hurst) exponent that indexes the long-range correlations in the structure of the noise across time scales where an exponent of .5 indicates white noise (which is movement that is random and follows no pattern), an exponent of 2 indicates brown noise (which indicates deterministic movement patterns), and importantly an exponent of 1 indicates pink noise (which indicates *some* relationship between movements at different time scales, so this movement is neither random, nor deterministic). Previous research has shown some indication that typically developing, healthy individuals show a motor movement scaling exponent in the pink noise ratio, that is neither random nor regular, but that shows flexibility [35]. Additionally, research has shown that brown or very regular and deterministic motion is associated with aging [37], Parkinson’s disease [38] and highly trained individuals like ballet dancers [64]. Due to these findings, it is in our interest to find the scaling exponent exhibited by children with ASD in their whole-body movements during a conversation to situate it in the larger research context.

Fractal analyses can provide us with a scaling exponent that best describes the variability in motion overall through a task, however time series can in fact visit several different scaling exponents at different times during the motion. MFDFA in turn measures the width of the distribution of all the possible exponents that exist in the variability due to intermittency in the dynamics underlying the interaction. In this analysis, a larger width suggests greater heterogeneity in the structure of long-range correlations across time scales. Even though multifractal analyses have only been used in the field recently, multifractality of body movements, specifically head movements, have been found during conversations [65].

Shuffled surrogate time series were used to determine whether the calculated fractal measures were significantly different from chance. By shuffling the time-series the amplitude spectrum and signal distribution of the experimental data is maintained, while the temporal relationships are broken, which then provides a measure of chance correlation levels. Two kinds of shuffled surrogate analyses were calculated, one for DFA and another for MFDFA. For DFA, random permutations were used to calculate surrogates from the time series [63]. For MFDFA analyses, surrogate data were generated using the iterative amplitude-adjusted Fourier transformation (IAAFT) method as described in [66]. The observed DFA exponents and MFDFA widths were subjected to statistical analyses to determine whether they were significantly different from those of the shuffled surrogate time series, and hence, whether there were significant long-term correlations in the data or width of the multifractal spectrum that would indicate interactivity between scales [67].

Finally, correlational analyses were used to determine if whole-body motor coordination and fractal structure of the motor signature were related to the severity of ASD (ADOS score) and the social cognitive abilities of the child. Both bivariate correlations and a principal components analysis were used.

For all statistical analyses, Greenhouse-Geisser adjustments for violations of sphericity were made as necessary. For post- hoc analyses, simple effect F-tests were used to analyze interactions and a Bonferonni criterion was used to determine significant differences between individual means.

## Results

**Fig 3:**
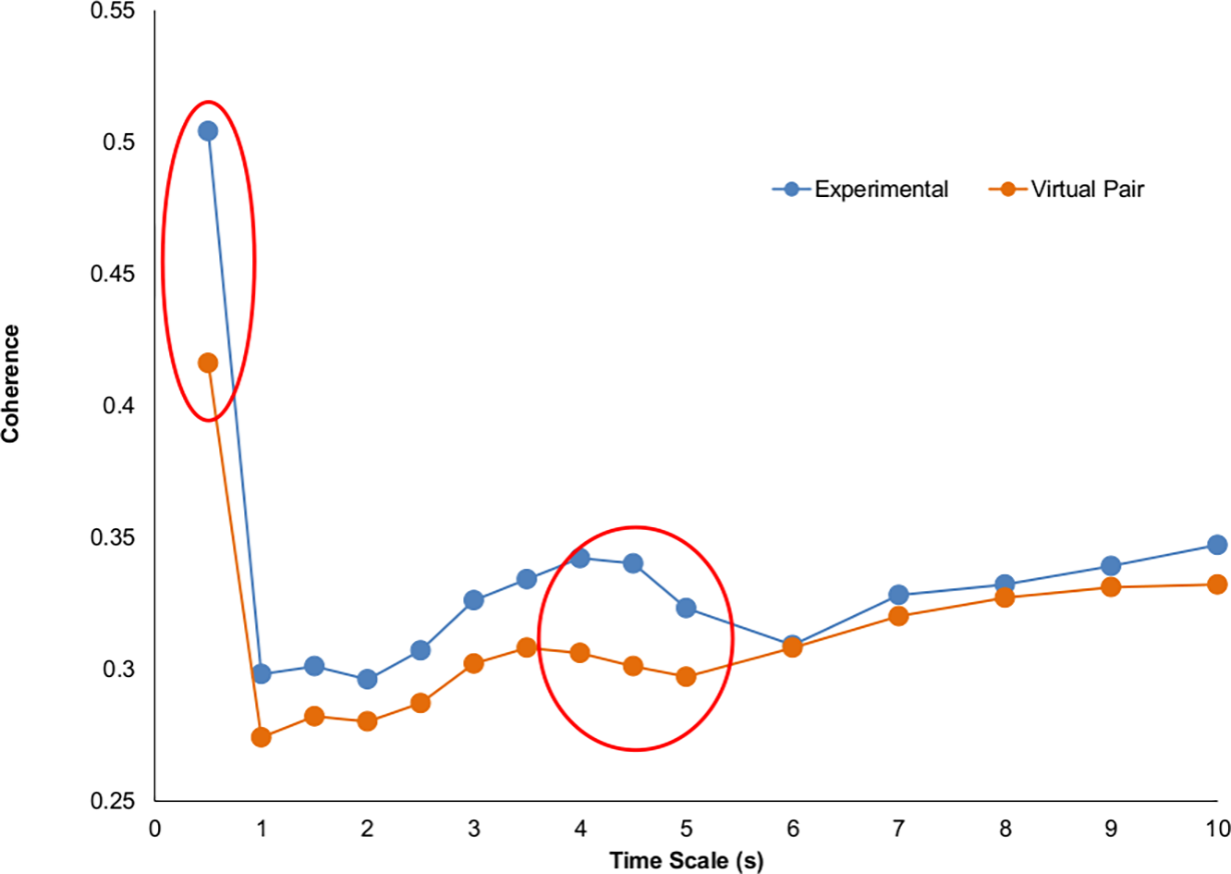
Synchronization between child and clinician. Degree of coordination of whole-body movements between the child and clinician at different time scales as measure by average wavelet coherence for experimental and virtual pairs (which represent chance coordination). Circled time scales represent where coordination was significantly above chance.

To evaluate whether the bodily movements of the child and clinician were coordinated, the average coherence at multiple time scales from the cross-wavelet analysis was submitted to a two-way ANOVA with within-subject variables of Condition (Experimental, Virtual) and Time Scale (0.5, 1, 1.5, 2, 2.5, 3, 3.5, 4, 4.5, 5, 6, 7, 8, 9, 10 s). Importantly, the analysis reveal a significant main effect of Condition (*F*(1, 27) = 4.33, *p* = .05, η_*p*_^*2*^ = .14) in which the coherence of the experimental pairs (*M* = .34) was found to be significantly greater than the chance coherence (*M* = .31) of the virtual pairs. The effect of Band (*F*(3.13, 27) = 27.04, *p* < .001, η_*p*_^*2*^ = .50) and the interaction of Condition and Band were both significant (*F*(3.05, 27) = 3.55, *p* = .02, η_*p*_^*2*^ = .12) as well. As one can see in Fig **3**, the experimental condition coherence values were greater than the virtual pairs for up until about the 5 s time scale. However, subsequent paired comparisons indicate that the experimental values were only significantly greater (*p* < .05) for the fastest time scale at .5 s and the 4.0 and 4.5 s time scales.

**Fig 4:**
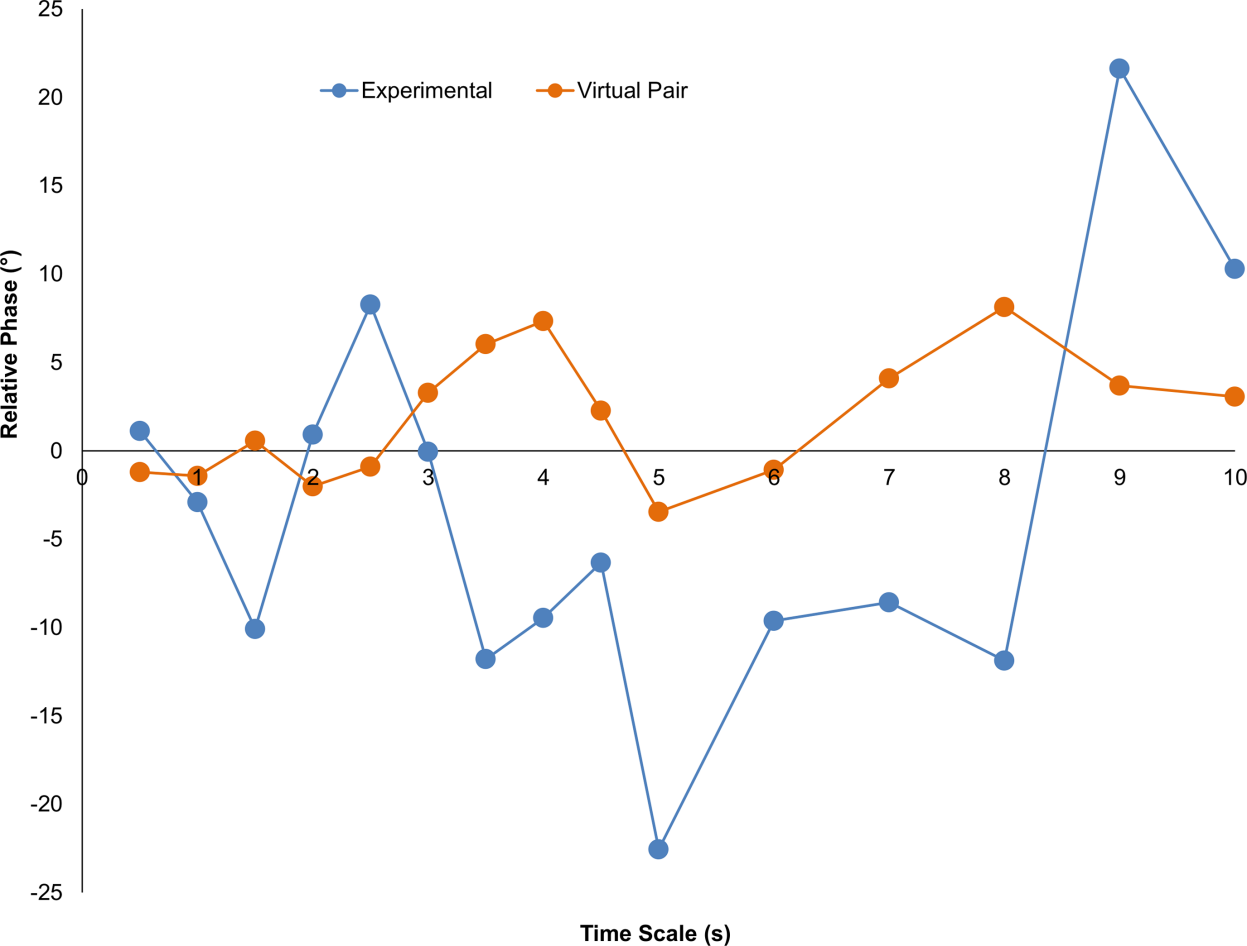
The relative phasing of whole-body movement rhythms of the child and clinician at the different time scales.

To evaluate *how* the bodily movements of the child and clinician were coordinated, that is, if there was a consistent lagging or leading of the child’s body movements during the interaction, the average relative phase angle at multiple time scales from the cross-wavelet analysis was submitted to a two-way ANOVA with within-subject variables of Condition (Experimental, Virtual) and Time Scale (0.5, 1, 1.5, 2, 2.5, 3, 3.5, 4, 4.5, 5, 6, 7, 8, 9, 10 s). The analysis revealed no significant effects. As shown in Fig 4 and verified by mean comparisons, the virtual pairs’ relative phase values were all near 0° signifying no lagging or leading and the experimental pair relative phase values did not significantly deviate from these chance values (*p* >.05).

**Fig 5:**
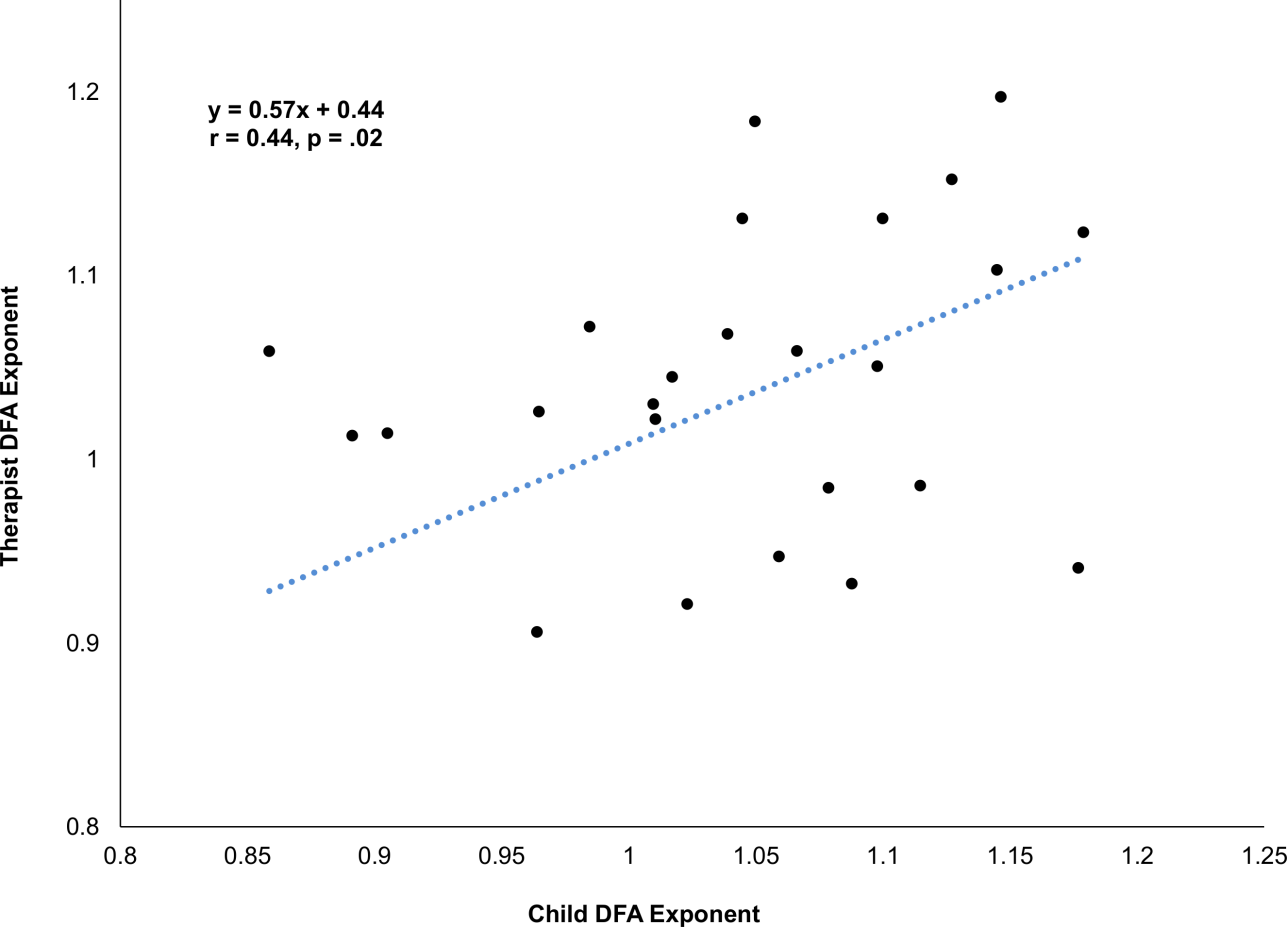
Correlation between fractal scaling exponents of child and clinician.

The DFA performed on both the child’s and the clinician’s whole-body movement time series yielded mean exponents close to 1 (Child: 1.041 (*SD* = .09); Clinician: 1.032, (*SD* = .11)), indicating pink noise (1/f) and long-term correlations in the structure of the variability. To evaluate whether child’s and clinician’s scaling exponents were significantly different from chance and from each other, they were submitted to a two-way within-subject ANOVA with variables of Condition (Experimental, Surrogate) and Role (Child, Clinician). A main effect of Condition (*F*(1,27) = 1755.0, *p* < .001, η_*p*_^*2*^ = .97) indicates that the bodily movements of the interviewer and interviewee both had a significant fractal structure (Experimental: 1.04, Virtual: 0.50). Neither the effect of Role (*F*(1,27) = 0.23, *p* > .05, η_*p*_^*2*^ = .01) nor the interaction of Condition and Role were significant (*F*(1,27) = 0.17, *p* > .05, η_*p*_^*2*^ = .01) signifying that both the clinician and child had a similar structure to their variability. Indeed, there was a significant correlation between the scaling exponents of the two interactors (*r*(26) = .43, *p* = .02; see Fig **5**) indicating what has been called complexity matching [68]—that there was a coordination of the structure of the variability of their movements across time scales.

The MFDFA performed on the activity time series suggested that the DFA scaling exponents appear to be multiple during the social interaction. The mean multifractal spectral widths were .35 (*SD* = .13) for the child and .37 (*SD* = .11) for the clinician. A two-way within-subject ANOVA with variables of Condition (Experimental, Surrogate) and Role (Child, Clinician) yielded a main effect of Condition (*F*(1,27) = 26.88, *p* < .001, η_*p*_^*2*^ = .50) in which the spectral width of the Experimental pairs (*M* = .36) was greater than the control condition surrogates (*M* = .30) indicating that the multifractal spectrums have widths significantly greater than expected by chance. Again neither the effect of Role (*F*(1,27) = 1.66, *p* > .05, η_*p*_^*2*^ = .06) nor the interaction of Condition and Role were significant (*F*(1,27) = 1.77, *p* > .05, η_*p*_^*2*^ = .06) signifying that the degree to which the clinician and child displayed intermittent dynamics and interaction across time scales was similar.

In order to determine whether the severity of ASD as well as the related social cognitive measures of joint attention and theory of mind deficits are embodied, that is, correspond to behavioral and dynamical indices of whole-body movements, correlational analyses were performed. First, bivariate correlations were performed evaluating the relationship of the dynamical measures (both child and clinician DFA and MFDFA measures as well as the ten coherence values indicating coordination up to the 5 s time scale) and the child’s total ADOS-2 score as well as their social affect (SA) and restricted and repetitive behavior (RRB) subscale scores [69]. As can be seen in Table 2, two dynamical measures of bodily movements were significantly correlated to the ADOS score but only for the RRB subscale, namely, the DFA scaling exponent of the child (*r* = 0.40) and the coherence between the bodily movements of the child and experimenter at the fastest time scale of 0.5 s (*r* = -0.40). As can be seen, the correlation between the DFA scaling exponent and the RRB score is positive, indicating that the more deterministic the child’s movement variability becomes, the higher RRB score they receive on the ADOS-2 test, which corresponds to previous findings in the literature regarding brown noise being associated with disorders [36]. Higher coherence between the clinician and the child’s movements on the other hand, is negatively related to the RRB score. This finding also is supported by previous research regarding the positive feedback loop that seems to exist between whole-body movement coordination among interacting individuals and other social measures [10–13], such that when children exhibited coordination with the clinician they tended to receive lower scores for repetitive behaviors in the ADOS-2 test.

**Table 2:** Bivariate correlations.

	ADOS Scale		
	Total	Social	Repetitive
**DFA Child**	0.21	-0.12	0.40*
**DFA Clinician**	0.18	0.05	0.31
**MFDFA Child**	0.08	0.02	0.11
**MFDFA Clinician**	-0.18	-0.02	-0.36
**Coherence at 0.5 s**	-0.20	0.04	-0.40*
**Coherence at 1.0 s**	0.08	0.24	0.07
**Coherence at 1.5 s**	0.04	0.16	-0.05
**Coherence at 2.0 s**	0.00	0.09	-0.04
**Coherence at 2.5 s**	-0.10	0.05	-0.10
**Coherence at 3.0 s**	0.12	0.18	0.01
**Coherence at 3.5 s**	0.15	0.05	0.05
**Coherence at 4.0 s**	0.18	-0.09	0.20
**Coherence at 4.5 s**	0.15	-0.16	0.23
**Coherence at 5.0 s**	0.01	-0.20	0.10

On the basis of these correlations, a principal components analysis was performed to see how cognitive measures of joint attention and theory of mind are related to these significant predictors of ASD severity, namely, ADOS severity score, and the Restrictive and Repetitive Behaviors (RRB) and Social Affect subscales (SA) as well as the movement measures. This analysis satisfied several adequacy criteria. First, all items correlated at least .3 with at least one other item, suggesting reasonable factorability. Second, the Kaiser-Meyer-Olkin measure of sampling adequacy was above the recommended value of .5, and Bartlett’s test of sphericity was significant. Additionally, the communalities were all above .5 confirming that each item shared some common variance with other items.

The analysis using varimax (orthogonal) rotation found two factors that explained 60% of the variance. The loadings of less than 0.40 were excluded. The results of this solution are shown in Table 3. A replication of the analysis using an oblimin (oblique) solution showed little difference. Four items, ADOS RRB subscale, theory of mind, Child DFA and Coherence at .5 s loaded onto factor 1, which explained 35% of the variance. Three items, ADOS SA subscale, theory of mind, and joint attention loaded onto a second factor that explained 25% of the variance.

**Table 3:** Results of the principal components analysis.

Item	Factor 1	Factor 2
**ADOS Social**		-.73
**ADOS Repetitive**	-.56	
**Joint Attention**		.72
**Theory of Mind**	.41	.56
**DFA Child**	-.90	
**Coherence at .5 s**	.88	

## Discussion

The current study was designed to better understand how whole-body movement coordination between a child with ASD and a clinician during a conversation is related to social problems associated with ASD. That is, how and whether the ASD social deficit is embodied. To this end, the study evaluated the degree of bodily movement coordination during conversation using a number of dynamical cross-wavelet measures of coordination. In addition, we tested the relationship between measures of social cognition and bodily movement coordination as well ASD severity as indexed by the ADOS-2 subscales. Given the role that social bodily coordination has been shown to play in social interactions in past research [8–13,33], relationships were expected to exist between these measures and the more traditional measures of ASD social cognition deficit and can be interpreted to provide an index of the degree of embodied cognition.

Cross-wavelet measures indicated that children with ASD coordinated their bodily movements with a clinician significantly more than what is expected to happen randomly. This finding is consistent with previous research which also found non-random coordination in teacher-student interactions [10], during joke telling [8,59], during brief friendly conversations [58], and while performing a series of tasks such as walking, rocking chairs, and cooperatively moving objects [9,12,13]. In fact, recent research has also found that children with autism are able to synchronize their body movements with others in a variety of tasks [18,28,30–32] however, their level of synchrony was below that observed in the control group. The current study lacked a control group and makes the comparison impossible. Future research should focus on this to further our understanding on the deficits observed in autism. Additionally, it was found that there was no consistent lagging or leading by the children, in other words, that the coordination found was happening simultaneously. Past research in healthy adults has found that participants do not synchronize their bodily movements during joke telling, rather the movement of one person leads the movements of the other [8]. However, given that [58] did observe this synchronization during brief, friendly conversations, the difference in findings might be driven by the type of conversational context being investigated. Future research involving a typically developing control group of children could test whether the differences observed are in degree of coordination or leader follower relationships since the current study cannot draw conclusions regarding this.

Further exploration of the bodily movements of the child and clinician found them to be fractal in the pink noise range and significantly more fractal than the surrogate random time-series. While different human behaviors have been shown to be fractal in nature in the past, such as gait [70–72], key pressing [73,74] and postural sway during standing [37,38,64], this is the first study in which whole-body movements during social interaction have been shown to be in the pink noise range. The fact that the coordination found between the child and the clinician was higher than chance indicates that these ASD children were indeed situated in the conversation. This result might be due to the nature of the conversation being had, in the sense that the child dictates what the topic of the conversation will be and is generally interested in discussing it. The fractal results indicate that the children in this sample showed healthy levels of motion variability with a scaling exponent around pink noise [36] rather than the highly regular motion that has been exhibited by adults with Parkinson’s disease [38], which is an encouraging result. Previous studies have shown that children with ASD exhibit higher levels of variability in their movement than typically developing [23,28–30,32], but these studies have not looked at the *type* of variability observed. Even though the results of the current study indicate that their variability is in the healthy range, without a control group to explore differences that might exist, we cannot reach conclusions as to the nuances that might be at play during conversations between clinicians and children with ASD. Furthermore, the current study only investigated one task of a highly structured diagnostic test (the ADOS-2) and the body movement coordination that arises between a child with ASD and a trained clinician, which could also explain the positive results found here. Additional research using other types of conversational exchanges between differing types of co-actors need to be investigated before firm conclusions can be drawn. Encouragingly though, these findings suggest that a more nuanced measure of conversational dynamics may be important for understanding the social problems that exist across the spectrum of individuals with ASD. Rather than focusing on *whether* an individual with ASD can pass a test to criterion, understanding the way in which the tasks are accomplished may provide important therapeutic insights.

Additionally, the complexity of the children’s movements matched the complexity of the clinician’s movements. Complexity matching research poses that people not only coordinate their movements and actions at local levels (as shown by the wavelet analysis in this study) but at different, nested time scales, which results in a matching of fractal exponents between actors [68,75]. Additionally, this complexity matching hypothesis has been found to result in an ability of actors to anticipate the motion of other actors or stimuli, even when the observed motions were chaotic [68,76,77]. More in line with the current study, complexity matching has been found to differentiate affiliative and argumentative conversations [75] and that the stronger the matching of complexity during a conversation, the more information is shared [78]. In the context of these previous findings, the current results therefore suggest that the children were (at least) partially engaged in the flow of information that defined the conversational exchange with the clinician. Of course, it remains unclear as to whether the observed complexity matching was due to the child alone, the clinician alone, or if both contributed evenly. Future studies could address this latter question by investigating the ability of children with ASD to exhibit complexity matching with a computer stimulus or avatar containing different levels of fractal movement variability. Unless we are able to fully control one of the sides of the interaction, we will not be able to understand if the complexity matching observed here is due to the clinician over-compensating for the child’s lack of coordination or if the children are able to exhibit this phenomenon as is expected of healthy people.

Finally, MFDFA measures revealed multiple scaling exponents during the conversation, and that the width of their multifractal spectrum was significantly larger than those expected by chance. In other words, children’s motions were found to be multifractal, which suggests greater heterogeneity in the structure of long-range correlations across time scales. This measure indicates that the complexity in their movement cannot be summarized by one fractal exponent because the system visits multiple states during the interaction. Previous research has found multifractality in quiet standing [79,80], gait [81], neuronal activation [82–84], and healthy human heartbeats [85]. The only other study investigating multifractal organization of movement in social behavior looked at the head movements of healthy adults during conversations [65] and their findings support ours, where adult head movements showed multifractal variability. Finding multifractality in the current study would indicate that the child and the clinician interact in a complex way during conversations, where the many components at play are related to each other dynamically and thus would indicate that in order to fully understand the phenomenon of conversing, it should be studied as a whole and not by isolating components. For conversations in particular, [65] suggest that multifractality in movement could also characterize the turn-taking situation that is part of a conversational exchange.

Next, we turn to the exploration of the relationship between bodily coordination and the ADOS-2 assessment test, which found that the dynamical structure of bodily movement (namely, the fractal nature of the child’s movement) and the whole-body movement coordination between the child and the clinician at a fast time scale, where people have been found to match bodily gestures such as laughing, smiling and nodding [61], to be related to the ADOS-2 restricted and repetitive behavior subscale. We found that the more deterministic the child’s movement variability, the higher RRB score they receive on the ADOS-2 test. In other words, children exhibiting more deterministic body movements were judged to be more impaired in terms of their RRB symptoms. This finding supports previous research which found that deterministic body movements were associated with disorders and more flexible movement (in the pink-noise range) was associated with healthy individuals [36]. The fact that our participants were higher-functioning individuals might account for the finding that we observed mostly pink-noise variability in their body movements. In the future, it would be interesting to study whether lower functioning individuals with ASD exhibit more deterministic movements. Additionally, higher coherence between the clinician and the child’s movement, was associated with lower scores for RRB in the ADOS-2 test. This result could support previous research findings regarding the positive influence that coordination between interacting individuals has on social measures [10–13]. Further exploration using principal components analysis found a relationship between the bodily movement measures and other traditional social cognition tools (joint attention and theory of mind). This analysis showed two components, the first comprised of the ADOS-2 RRB subscale, theory of mind, child DFA and coherence between child and clinician’s bodily movements at the 0.5 second time band; the second factor was comprised of the ADOS-2 SA subscale, theory of mind and joint attention.

The dynamical activity measures loaded on to the first factor along with the ADOS-2 index of restricted and repetitive behaviors (RRB) which include the preoccupation with restricted patterns of interest, adherence to specific, nonfunctional routines, repetitive motor manners and preoccupation with parts of objects [86]. One could see how such behaviors could be related to bodily movement patterns in a social interaction. As explained by [87], there are two different RRB factors: repetitive sensory-motor behaviors and insistence on sameness. In this case, we are especially interested in the insistence on sameness (IS), which is manifested by repetitiveness with respect to conversational topics and resistance to change [87–89]. Specifically, Cuccaro and colleagues [89] see this measure as “The individual (is) imposing an order (or responding to a lack of order) in the environment” (p. 13). In this case the increase in RRB measures seems to be associated with an increase in the degree of long-term autocorrelations in the activity of the child (Child’s DFA measure), which is generally taken to correspond to an increase in control [36], as well as a decrease in the synchronization of the child’s activity with the clinician. This result provides concrete evidence for observations that have been made in the literature many times, where children with ASD show insistence of sameness, which in this case equates to the increase in control [1,89]. Furthermore, the current results show that this need for control is manifested not only in the verbal content of the conversation but also in the non-verbal, subtle aspects of social communication. Also included in this factor is the theory of mind measure—a measure of social knowledge the child has about others, specifically the extent to which the child can take the perspective of another to understand their motives. This result verifies past research that has demonstrated that higher levels of social synchronization in children with ASD is associated with higher ability in understanding another’s mental life [28]. It may be that the lack of awareness of other mental agents that is associated with the restricted and repetitive behaviors of ASD increases the intrinsic degree of correlation in a child’s activity that then does not allow for a flexible coordination with another person in a social interaction. Children then may be less able to coordinate with another person due to their lack of flexibility, which makes it harder for them to build rapport [10–13] and in turn manifests itself as a deficit in theory of mind known to be part of ASD [40,41,44,45]. Importantly, the fact that the dynamic whole-body movement measures are related to higher social cognition measures indicates that these high functioning children with ASD are showing some degree of embodied social competence in the sense that the level of their social cognition seems to manifest itself also in their whole-body movements during this type of interaction. Additional research is needed to evaluate the relationship between these measures across a larger spectrum of ASD severity and to determine whether these processes share underlying mechanisms or whether one contributes to difficulties in the other.

For the second factor in the principal components analysis, we see the traditional measures of social cognitive deficits (theory of mind and joint attention) load with the social affect ADOS-2 subscale. This subscale indexes the frequency of typical social behaviors such as the use of gestures, making eye contact, and appropriateness of responses during social interactions [69]. This factor is devoid of the dynamical whole-body movement coordination measures, however. It is perhaps not surprising that the dynamical measures do not correlate with this scale since they correspond more to the *content* of a social interaction rather than the *manner* in which it might unfold in time. This factor then captures the diminished capacity of children with ASD in terms of conversational content during social interactions [90–94].

While some of the findings of the current study are interesting and supported by previous research, it was not without its limitations. First, the sample was comprised of high functioning children with ASD and future research would benefit from investigating these measures in a wider range of ASD severity. Most importantly, the current study did not have access to a control group of typically developing children because these children did not complete an ADOS-2 evaluation. Additionally, ADOS-2 scores were assigned by the clinician that administered the assessment and could therefore possibly confound some of the results reviewed, especially the factor analysis. This is unlikely given that the clinician was trained to research–reliability in administration of the ADOS, a very stringent criteria to ensure consistency in administration and scoring, the clinician was blind to the fact that bodily coordination measures would be made from the ADOS session, and the bodily coordination measures were calculated in an automated fashion (which eliminates the possibility of subjective biases inherent in behavioral coding). One way in which this could be mitigated in the future is by asking other trained clinicians to observe the clips of the assessment and provide another rating that can then be compared to that of the administrator. Future research should also look at the coordination measures exhibited by children in a control group performing a similar task to further understand what part social motor coordination plays in the ASD impairment. The current study found above-chance bodily coordination between the child and the clinician, but without a control group we cannot know if the differences will be of degree of coordination (with typically developing children coordinating more) or due to the fact that the pattern of coordination observed might be different. As mentioned previously, synchronization emerges as the result of the interaction between two people. It is possible that the degree of synchronization observed is the consequence of the therapist matching the movement of the child, rather than an equal bi-directional coupling [31]. Analysis of bodily movement coordination between peers (with and without) ASD would shed light on this issue, as would comparison of bodily coordination between conversational partners communicating remotely with and without visual information.

## Conclusion

In conclusion, the current study provided evidence that children with high functioning autism spectrum disorder are capable of coordinating their body movements with a clinician during a conversation in a manner that is above chance levels. This suggests they are embodying *some* degree of social competence during conversations with clinicians. Furthermore, our preliminary evidence suggests that the degree of this bodily coordination is related to social cognitive ability. Taken together, this demonstrates the importance of further investigating the subtle but important bodily movement coordination that develops during social interaction in general and in children with ASD specifically. These objective measures of conversation dynamics have the potential to provide a deeper understanding of the social cognition impairments known to be a central part of autism spectrum disorders.

## Appendix A: Transcripts of sample conversations

**T**: **Therapist/Test Administrator**

**C**: **Child/Participant**

### Example 1 of conversation task

T: Have you ever been fishing?

C: Yeah but we never catch anything

T: Oh

C: And some random drunk lady probably going waterskiing and then there’s a some kid who’s stuck in a tire and then I see trees and a people and some lady and an old lady in a car

T: You know I used to hate going fishing with my grandfather

C: And then some old person trying to get a hole in one

T: Because he used to make me bait a hook by sticking a hook through the crawfish’s head

C: Oh

T: Mhmm I didn’t like that at all. And one time I went fishing and guess what happened?

C: What?

T: Well I went out on a big big lake on a big big boat and we got way far away from the shore and there was a huge storm came and a really bad thing almost happened

C: What

T: The boat almost tipped over

C: Man

T: Uh-huh we had to wear life vests and everything

C: When we go fishing we um we uh we always going on the um on the um pavement we always stand on the pavement

T: Oh ok

C: Then we use the, anyhow fishing’s more entertaining unless I’m on a game called Minecraft. It’s this game where you have this little character and then

T: Oh I’ve played Minecraft before

C: Oh

T: Uh-huh

C: I play it

T: I’ve only seen I’ve only seen like one of the levels on Minecraft though.

C: Yeah there’s uh, there’s like a couple different ones. First there’s peaceful, where you heal automatically and there’s no monsters coming out on easy and they only come out at night but they’ll do a little damage

T: Uh-huh

C: And then there’s normal where uh enemies come but they do a slight amount more damage. On hard lots of enemies spawn on the environment, and they do a great amount of damage. There’s also creative too.

T: Now I don’t understand on Minecraft how time passes.

C: Well yeah um it’s just like the real world um like with the sun it goes up in the sky and then it goes down and then it becomes night for a little bit then it uh comes morning again and then if you’re on another difficulty then zombies and skeletons burn up and die, unless they go under the shade or water.

T: I haven’t seen the zombies and skeletons.

C: Yeah they’re found on easy and on hard.

T: Ok

C: And there’s a hardcore where you get one life and then it’s locked on the hardest difficulty, it’s hardcore. And you only get and if you die, you really die so it makes it a little more realistic.

T: Ok and I assume the zombies and the skeletons are kind of blocky like the rest of the.

C: Yeah it’s kind of like I’m guessing either 64, maybe like um a 16 bit game or something like that

T: It looks so old like you know is the interesting thing about Minecraft is that it looks like it was made for many many years ago not for today

C: Yeah like I think it was made in 2009 like as a virtual game where you just go around destroying like there wasn’t any enemies or anything and then uh there’s this video um just go to Youtube like um go to Youtube and then type in the evolution of Minecraft and then look for one of them will give you information of how it began and how it was

T: Ok

C: Yeah

T: Wow

C: Yeah that’s how it’s been

### Example 2 of conversation task

T: Wow. Have you ever been to the ocean?

C: Um I think so.

T: You think so? Hmm, have you ever been on vacation?

C: Yes.

T: Tell me about your favorite vacation

C: Um, I think uh this is one of my only ones

T: Ok

C: North Ca- North Carolina, um, well, well went there well, we went there go see some dolphins in a boat

T: Mm-hmm, wow that sounds neat

C: Mm-hmm. And we went to the boat and I think it was the ocean

T: Uh-huh

C: Hmm

T: You know I’ve never seen dolphins before, ever.

C: Wow

T: Mm-mm. I bet it was amazing.

C: Mm-hmm

T: Uh-huh. What else did you do?

C: Um, let’s see, ok so we also went inside of a store and each of us bought something well I bought, I bought a snow globe and

T: Mm-hmm

C: And it was like a little souvenir

T: (laughs)

C: With a lighthouse and we also went to a lighthouse

T: You did?

C: Mm-hmm. Well my little sister and my friend’s little sister were too young to go up the lighthouse, but me, but me and my friend were old enough.

T: You got to go up in the lighthouse?

C: (nods)

T: Oh my goodness.

C: And there was a great view at the very top.

T: And usually there’s a light in the lighthouse, right? And sometimes the light works but sometimes the light doesn’t work.

C: Hmm, which reminds me, I have a light I have a little lighthouse at home with four books in it.

T: Four books inside the lighthouse?

C: Uh-huh

T: I don’t understand

C: Well, miniature version I think

T: Oh so it’s a statue.

C: Kind of, kind of not.

T: Not?

C: Well, and I think the, I think the the light in in the tiny lighthouse

T: Mm-hmm

C: Well I think it used to play a song and twirl around

T: Oh, but it doesn’t do that anymore

C: No.

T: The lighthouse holds the books standing upright?

C: (nods)

T: Oh, it’s a bookend

C: Mm-hmm

T: That’s really cool. I don’t have any bookends that look like lighthouses but I have been to a lighthouse, yah, and when I was there something really awesome happened

C: Wow

T: It really kind of, it really almost kind of scared me a little bit.

C: Wow.

T: And it could have been dangerous. Do you want to know what it was?

C: (Nodding excitedly)

T: So I was up in the lighthouse and the lighthouse was up at the very very end of this long walkway in the middle of the ocean and this huge wave came up and hit the window of the lighthouse and for a while I thought it was going to wash us away.

C: Yikes

T: I know, yikes, that’s right. Ok. What else do you see?

C: Um an airplane.

T: Have you ever been in an airplane?

C: Um when I was when I was a baby

T: Oh, do you remember it?

C: No. Ok, so someone is holding a bra while floating.

T: (laughs) that’s silly, that’s really silly. What else do you see?

C: Let’s see, um, well well someone has a snor- a snorkel

T: Mm-hmm

C: And they’re up, a and they’re above the water.

T: Oh, do you know how to swim?

C: Yes.

T: Yes? Tell me about when you learned how to swim.

C: Oh. YMCA.

T: Oh, you went to one of the big pools in the YMCA?

C: Um, no swim lessons.

T: Oh you got to have swim lessons. You know I didn’t learn at a YMCA, my mom taught me how to swim. And she taught me how to swim in the ocean

C: (gasp)

T: I know, and it was a little bit scary because the waves kept coming and going up and down and I had a hard time staying afloat.

C: um, and it’s it’s always a good idea to stay in the shallow end of of well well if there’s water in, of course in the ocean and I think there is an ocean on the beach in Nor- in North Carolina

T: Mm-hmm, where you went on vacation

C: Yes

T: Mm-hmm

C: Well well it was a good idea just to stay in the shallow end.

T: Yeah because the Atlantic Ocean is a big ocean.

C: And plus there would be sharks and jellyfish.

T: Oh there could be sharks and jellyfish in there and I don’t know about you, but the salt in the water, sometimes it gets in my mouth and tastes kind of yucky.

C: Yep, and and it got in my eyes.

T: It got in your eyes too? Oh, better go and wash your eyes out if it gets in your eyes. Although did you know that there is salt in your eyes?

C: Wow

T: Mm-hmm, our body has lots of salt in it. MM-hmm, alright.

C: And de and oh it’s only dead jellyfish that wash up

T: Oh because the rest of them are swimming in the ocean

C: Mm-hmm.
