# Correction: Evidence of embodied social competence during conversation in high functioning children with autism spectrum disorder

**DOI:** 10.1371/journal.pone.0195888

**Published:** 2018-04-10

**Authors:** Veronica Romero, Paula Fitzpatrick, Stephanie Roulier, Amie Duncan, Michael J. Richardson, R. C. Schmidt

The following information is missing from the Funding section: National Institute of Mental Health also provided funding under award number R01GM105045 (MJR, RCS).
